# Developing Allosteric Chaperones for *GBA1*-Associated Disorders—An Integrated Computational and Experimental Approach

**DOI:** 10.3390/ijms26010009

**Published:** 2024-12-24

**Authors:** Marta Montpeyo, Natàlia Pérez-Carmona, Elena Cubero, Aida Delgado, Ana Ruano, Jokin Carrillo, Manolo Bellotto, Marta Martinez-Vicente, Ana Maria Garcia-Collazo

**Affiliations:** 1 Neurodegenerative Diseases Research Group, Vall d’Hebron Research Institute (VHIR)—Network Center for Biomedical Research in Neurodegenerative Diseases (CIBERNED), 08035 Barcelona, Spain; marta.montpeyo@vhir.org; 2 Gain Therapeutics Sucursal en España, Parc Científic de Barcelona, 08028 Barcelona, Spain; nperez@gaintherapeutics.com (N.P.-C.); ecubero@gaintherapeutics.com (E.C.); adelgado@gaintherapeutics.com (A.D.); aruano@gaintherapeutics.com (A.R.); jcarrillo@gaintherapeutics.com (J.C.); 3 GT Gain Therapeutics SA, 6900 Lugano, Switzerland; mbellotto@gaintherapeutics.com

**Keywords:** *GBA1*, glucocerebrosidase, pharmacological chaperone, Gaucher disease, Parkinson’s disease, lysosomal storage disease, lysosome

## Abstract

Mutations in the *GBA1* gene, which encodes the lysosomal enzyme glucocerebrosidase (GCase), are associated with Gaucher disease and increased risk of Parkinson’s disease. This study describes the discovery and characterization of novel allosteric pharmacological chaperones for GCase through an innovative computational approach combined with experimental validation. Utilizing virtual screening and structure-activity relationship optimization, researchers identified several compounds that significantly enhance GCase activity and stability across various cellular models, including patient-derived fibroblasts and neuronal cells harboring *GBA1* mutations. Among these, compound **3** emerged as a lead candidate, demonstrating the ability to enhance GCase protein levels and enzymatic activity while effectively reducing the accumulation of toxic substrates in neuronal models. Importantly, pharmacokinetic studies revealed that compound **3** has favorable brain penetration, indicating its potential as a disease-modifying therapy for *GBA1*-related disorders affecting the central nervous system. This research not only offers a framework for developing allosteric GCase modulators but also unveils promising new therapeutic strategies for managing Gaucher disease and Parkinson’s disease. The ability of compound **3** to cross the blood-brain barrier emphasizes its potential significance in addressing neurological symptoms associated with these conditions.

## 1. Introduction

Biallelic mutations in the *glucosylceramidase beta 1* (*GBA1)* gene cause Gaucher’s disease (GD), an autosomal recessive disease and the most prevalent lysosomal storage disorder (LSD) [1,2,3]. The *GBA1* gene encodes the lysosomal enzyme β-glucocerebrosidase, also known as GBA or GCase. As a lysosomal hydrolase, GCase is synthesized in the endoplasmic reticulum (ER) and, aided by its transporter, lysosomal integral membrane protein-2 (LIMP-2), is transported through the Golgi apparatus to its destination at the lysosome [4]. This hydrolytic enzyme is essential for sphingolipid metabolism, converting glucosylceramides (GlcCer) into glucose and ceramide within the lysosome [5]. Variants of *GBA1* associated with reduced GCase activity can cause accumulation of the substrate GlcCer and alter numerous other downstream products in the sphingolipid pathway, including GlcSph, the deacetylation product of GlcCer, also known as LysoGb1, which is a key biomarker for diagnosis and monitoring GD [2,6,7,8]. Moreover, heterozygous *GBA1* variants represent the most prevalent genetic risk factor for alpha-synucleinopathies such as Parkinson’s Disease (PD) and Dementia with Lewy Bodies (DLB) [9,10,11]. This association underscores the important interplay between lysosomal dysfunction and neurodegeneration [12].

Current strategies for managing GD include enzyme replacement therapy (ERT) and substrate reduction therapy (SRT) [13]. ERT was the first treatment developed for GD, which involves intravenous infusion of recombinant GCase to replace the deficient enzyme [14]. Although ERT can be effective in reducing and reversing clinical systemic symptoms like visceral organ enlargement and improving haematological parameters, it is ineffective against neurological symptoms. This limitation exists because recombinant proteins are unable to penetrate the blood-brain barrier (BBB). SRT involves the oral use of small molecule inhibitors that target glucosylceramide synthase to decrease the production of glucocerebroside, a substrate of GCase. This approach aims to prevent the buildup of GlcCer and GlcSph/lysoGb. However, current SRT options have minimal ability to penetrate the BBB, leading to limited effectiveness in addressing neurological symptoms in GD patients [15,16]. In addition, SRT is a therapy focused only on preventing sphingolipid accumulation, while ERT and other emerging therapies aim to restore the enzyme’s activity, which could have a broader therapeutic impact, improving overall lysosomal function and mitigating other effects of the enzyme deficiency. One such emerging therapeutic approach involves pharmacological chaperones (PCs). These are small molecules capable of binding to mutant GCase, facilitating its proper folding, stabilizing it to prevent degradation, and enhancing its transport to the lysosome, thereby reducing substrate accumulation. Although the majority of PCs developed to date are inhibitory and bind to the enzyme’s active site [17,18,19], there is a growing interest in developing allosteric chaperones. Allosteric chaperones offer a promising therapeutic alternative as they do not compete with the enzyme’s natural substrate or inhibit its activity.

The SEE-Tx^®^ computational drug discovery platform has facilitated the discovery of small molecule structurally targeted allosteric regulators (STARs) [20,21]. By leveraging three-dimensional protein structures alongside our advanced supercomputer-powered methods, this physics-based screening technology aims to uncover potential druggable allosteric sites, thereby aiding in the selection and validation of PCs. Through our proprietary virtual screening procedure, GCase STARs, which have potential as disease-modifying therapies for the treatment of GD and PD, were discovered. This paper outlines the process of screening, identifying, and experimentally validating compounds capable of stabilizing the GCase enzyme, improving its activity, and reducing sphingolipid accumulation. This work highlights the complexity of designing and validating an experimental approach to identify a GCase-specific allosteric pharmacological chaperone.

## 2. Results

### 2.1. Identification of GCase Small Molecule Stabilizers (STARs) Using SEE-Tx Supercomputing Technology

Gain’s SEE-Tx^®^ drug discovery approach identified a novel druggable allosteric cavity in the high-resolution structure of native GCase (PDB code: 2V3F) [22]. The potential of this allosteric pocket to bind small molecules was assessed using MDmix v0.1 [23,24,25,26,27], confirming its suitability for virtual screening with rDock 2013.1 software [28].

**Figure 1:**
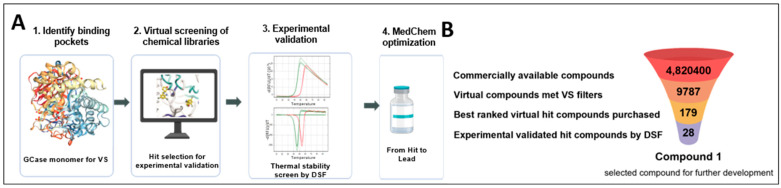
The SEE-Tx^®^ approach to discover non-competitive, pharmacological allosteric regulators for the GCase protein: (**A**) Schematic workflow of the procedure used to discover new allosteric regulators; (**B**) Docking-based high-throughput virtual screening of small molecules. Abbreviation: GCase; Glucocerebrosidase; VS, virtual screening; DSF, differential scanning fluorometry.

Our screening methodology efficiently filtered through an extensive virtual library of approximately five million non-redundant compounds. As a result, the original library was reduced to 9787 small molecules that met the standard scoring function, stringent pharmacophoric constraints, and high-throughput protocol. A set of 179 top-ranked diverse molecules were selected for further experimental evaluation. These molecules were screened using differential scanning fluorimetry (DSF) to assess their ability to stabilize the recombinant human GCase protein (Figure 1A,B).

DSF is a method used to evaluate protein thermal stability and detect ligand interactions by observing how binding affects protein stability. Specifically, it examines the interaction between small molecules and the human GCase protein. DSF uses fluorescent dyes to track protein unfolding as temperature changes. As the protein unfolds due to rising temperatures, its hydrophobic regions become exposed, altering the dye’s fluorescence signal. This change helps determine the melting temperature (Tm). Variations in Tm compared to the protein alone reveal interactions with tested compounds, aiding in identifying molecules that stabilize GCase.

**Figure 2:**
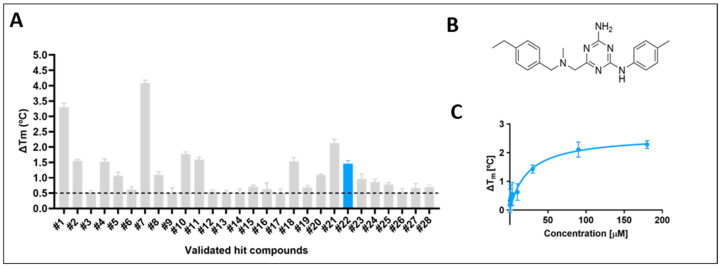
Binding of small molecule hit compounds to GCase, as determined by DSF: (**A**) Difference in melting temperature (ΔTm) relative to recombinant human GCase in the presence of hit #1 to #28 at 30 μM and pH 7.2. The mean ΔTm values ± SD are from 2 independent experiments (*n* = 2). The dotted line shows the threshold value for the DSF screening, which was ΔTm ≥ 0.5 °C; (**B**) Compound **1** (hit #22) structure; and (**C**) Dose-dependent effect on the thermal stability of GCase in the presence of hit #22 (compound **1**). The mean ΔTm values ± SD are from 2 independent experiments (*n =* 2). DSF, differential scanning fluorimetry; GCase; glucocerebrosidase; SD, standard deviation; Tm, melting temperature.

From the initial screening, 28 virtual hit compounds underwent experimental validation, demonstrating improved thermal stability of the GCase protein as measured by DSF (Figure 2A and Table S1). Most compounds exhibited a shift in melting temperature (Tm) compared to the baseline, indicating protein stabilization. Notably, hit #22, now referred to as compound **1**, showed a Tm shift of 1.45 °C degrees (Figure 2B). This compound established a new chemical scaffold that serves as the basis for a medicinal chemistry program aimed at developing GCase stabilizers that act as PCs. While some compounds from the initial set of validated hits demonstrated greater thermal stabilization than hit #22, they were eliminated for reasons such as being singletons, exhibiting multifluorescence, high lipophilicity, instability, or being classified as frequent hitters. Ultimately, compound **1** was identified as the most promising due to its drug-like characteristics, making it a valuable candidate for further development. The effect of binding on the stability of recombinant human GCase was also measured with increasing doses of compound **1** at pH 7.2 (Figure 2C). This DFS analysis revealed a binding affinity (K_D_) of 23.6 μM.

### 2.2. Experimental Validation of STAR Compound: Effect of Compounds on Enhancing GCase Activity in Patient-Derived Fibroblasts

The effect of the identified compounds on intracellular GCase activity was assessed using a GCase activity assay. This evaluation was conducted using fibroblast cell lines derived from healthy controls and GD patients with genotypes p.L444P/p.L444P, p.N370S/84gg and p.N188S/p.S107L. Additionally, a PD patient with the genotype p.L444P/p.WT was included in the study. The assay served as a primary screening tool to aid the development of subsequent compounds and guide medicinal chemistry efforts. In this experiment, cells were exposed to the compounds for four days, followed by cell lysis. GCase activity was measured using the artificial fluorogenic substrate 4-methylumberlliferyl-β-D-glucopyranoside (4-MUG) [29]. Initially, compounds were screened at a concentration of 12.5 µM.

**Figure 3:**
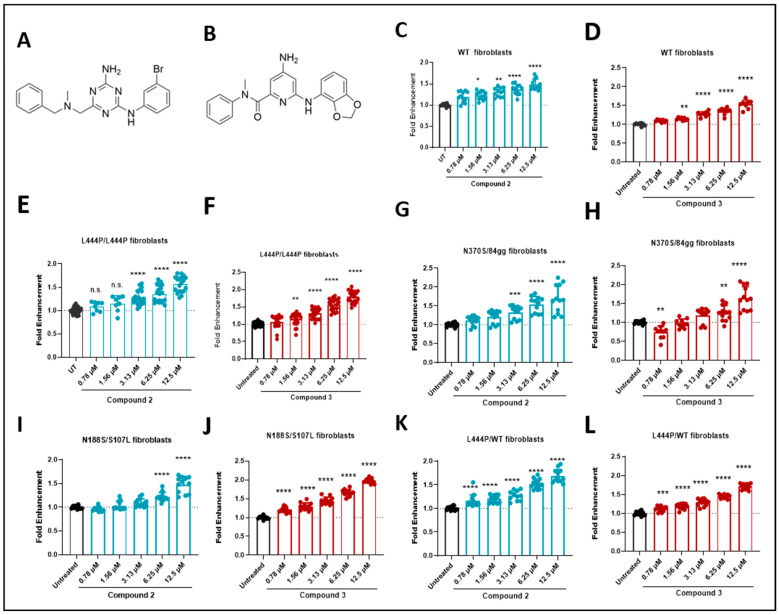
Validation of STAR compounds: (**A**) Structures of compound **2**; (**B**) structure of compound **3**; and measurement of GCase activity in WT fibroblasts treated for four days with (**C**) compound **2** and (**D**) compound **3**; and *GBA1*-associated patient-derived fibroblasts treated for four days with compound **2** and **3**, respectively: (**E**,**F**) p.L444P/p.L444P; (**G**,**H**) p.N370S/84gg; (**I**,**J**) p.N188S/p.S107L; and (**K**,**L**) p.L444P/p. WT. Mean values from at least three replicates of three independent experiments are represented by bars. Results are normalized to the untreated and presented as mean ± SD values of three experiments after one-way ANOVA with Dunnett’s multiple comparison test, * *p* < 0.05, ** *p* < 0.01, *** *p* < 0.001, **** *p* < 0.0001; n.s.—no significant; WT, wild-type.

Among the experimentally validated virtual hits tested in the enzyme enhancement assay using fibroblasts, compound **1** showed significant potential by increasing the GCase activity by 1.8-fold in p.L444P/p.L444P fibroblasts. This promising result initiated the medicinal chemistry development of a chemical series based on its chemical scaffold. The structure-activity relationship (SAR) analysis focused on the aniline and benzylamine components of the original compound **1**, leading to the development of compound **2**, which served as a tool compound during the hit-to-lead stage (Figure 3A). Further refinement of the chemical series involved introducing a carbonyl group and using pyridine as the core structure to improve its ADME-Tox properties (absorption, distribution, metabolism, excretion, and toxicity). These modifications specifically improved metabolism in mouse liver microsomes and reduced hERG inhibition, resulting in the development of compounds such as compound **3**, a key representative of this lead series (Figure 3B). Selected compounds **2** and **3** effectively increased GCase activity in a dose-dependent manner across all fibroblast samples, regardless of the *GBA1* genotype (Figure 3C–L).

### 2.3. Binding of Compounds to GCase and Effect on Enzymatic Activity

**Figure 4:**
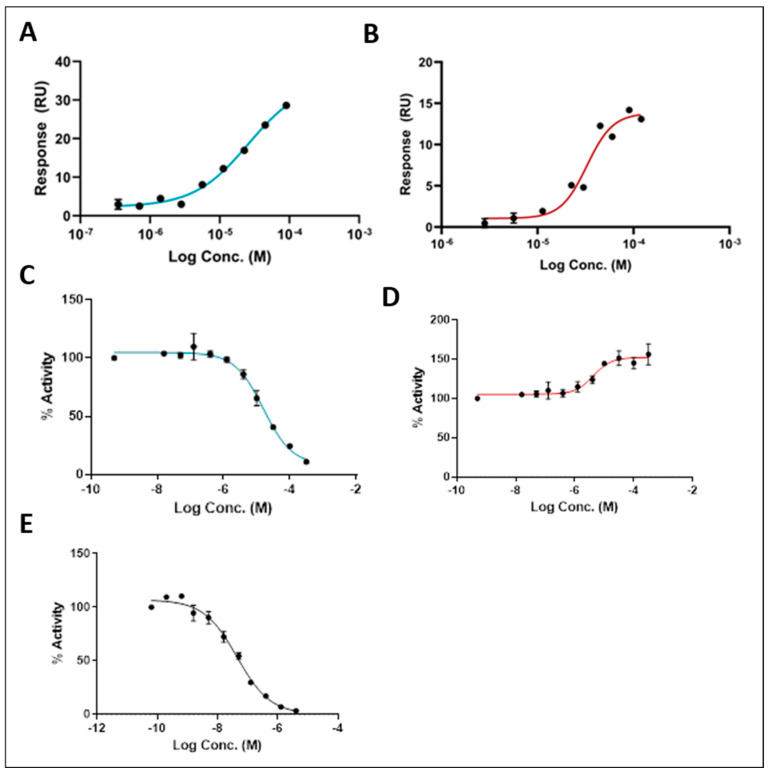
Dose-response for: (**A**) compound **2** and (**B**) compound **3** binding to immobilized human recombinant GCase monitored at neutral pH (7.4) by SPR; and GCase activity assay in wild-type lysates after treatment with (**C**) compound **2**, (**D**) compound **3,** and (**E**) isofagomine (IFG) at acidic pH (5.6). Dose-response curves are plotted with mean values based on two independent assays with three replicates each. Error bars represent the standard deviation of the means.

The surface plasmon resonance (SPR) technique is used to detect changes in the refractive index on the surface of a sensor due to changes in mass. These changes were used to quantify the biomolecular interactions between the purified GCase protein and our lead compounds [30,31]. Binding to GCase by SPR was confirmed for selected compounds using recombinant human GCase protein at a neutral pH of 7.4 (Figure 4A,B). Compounds **2** and **3** showed a binding affinity (KD) of 29.8 and 50.7 µM, respectively, confirming their ability to bind to the target GCase protein (Table 1). Compounds **2** and **3**, along with isofagomine (IFG) as a control, were evaluated in an activity assay using lysates from wild-type fibroblasts (Figure 4C). Despite the SEE-Tx^®^ technology revealing new allosteric sites on GCase beyond the active site, compound **2** displayed inhibitory effects. In contrast, compound **3** was identified as a neutral binding chaperone, also known as a silent allosteric modulator, since it improved cellular activity by stabilizing the GCase protein without altering its enzymatic function. As anticipated, IFG demonstrated an inhibitory effect (Figure 4C).

### 2.4. Effect of Compounds on Lysosomal GCase Activity and Substrate Depletion

**Figure 5:**
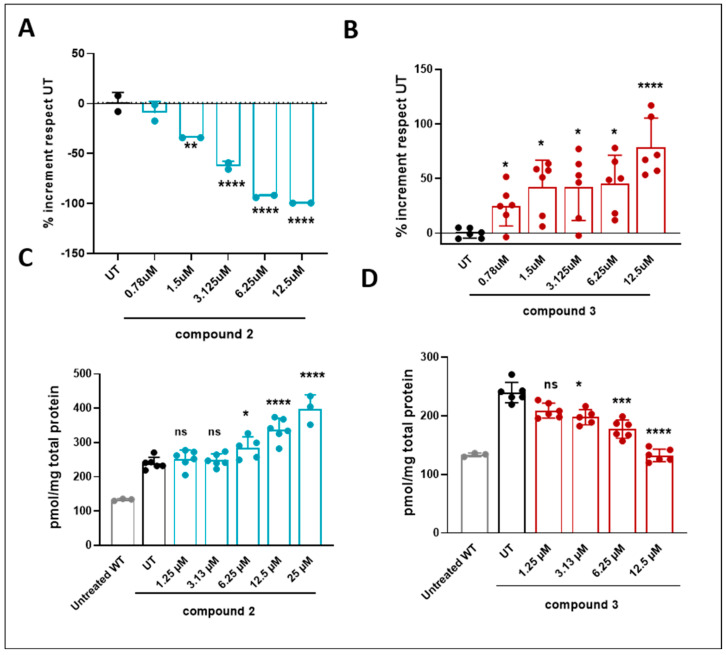
Measurement of GCase activity in WT cell lines and fibroblasts derived from GD patients treated for four days. Mean values were derived from (**A**) one independent experiment with two replicates for compound **2** and (**B**) three independent experiments with two replicates each for compound **3**. HexCer substrate quantification to evaluate the substrate depletion in GD patient-derived fibroblasts (L444P/L444P) by LC/MS-MS for (**C**) compound **2** and (**D**) compound **3**. Results are presented as mean values from three replicates of two independent experiments after one-way ANOVA with Dunnett’s multiple comparison test, * *p* < 0.05, ** *p* < 0.01, *** *p* < 0.001, **** *p* < 0.0001; ns, no significant; WT, wild-type.

To validate the observed chaperoning effect of the compounds, an in situ lysosomal GCase assay was conducted using a fluorescent PFB-FDGlu probe in fibroblasts from GD patients carrying the p.L444P biallelic mutation. Consistent with earlier findings (Figure 4C), compound **3** demonstrated a dose-dependent enhancement of lysosomal GCase activity. Conversely, compound **2** exhibited an inhibitory effect that intensified with increasing concentrations, aligning with its previously observed inhibitory behavior (Figure 5A).

L444P/L444P fibroblasts have impaired GCase activity due to the presence of the mutation, resulting in the accumulation of toxic substrates, glucosylceramide (GlcCer), and other related sphingolipids in the lysosome [32,33,34]. To evaluate the efficacy of the compounds in reducing substrate accumulation, we quantified monohexosylceramide (HexCer) species (including GluCer) after treatment of L444P/L444P fibroblasts with compounds **2** and **3**. The accumulation and clearance of these substrates were measured by liquid chromatography-tandem mass spectrometry (LC/MS-MS), quantifying the total HexCer species [35,36]. In line with previous findings on lysosomal GCase activity, compound **2** was ineffective in lowering sphingolipid levels. Moreover, at higher concentrations, it inhibited GCase activity (Figure 5A), leading to increased accumulation of HexCer (Figure 5C). On the other hand, compound **3**, a non-competitive agent, enhanced GCase activity (Figure 5B) and successfully decreased HexCer levels (Figure 5D).

### 2.5. Enhancement of GCase Activity and Depletion of Toxic Substrate in a Neuronal Model

**Figure 6:**
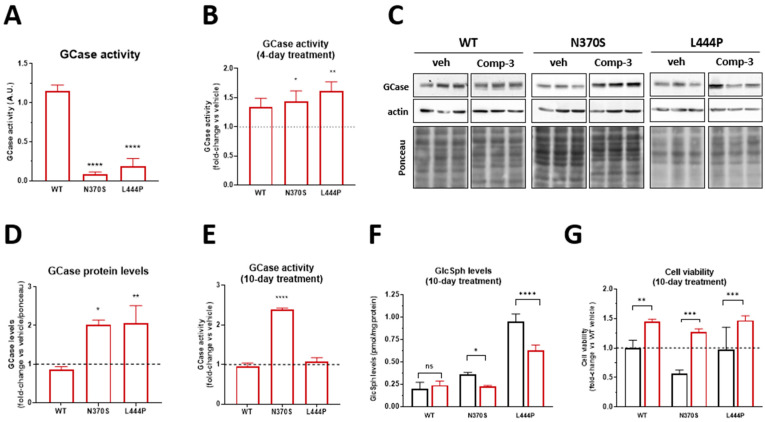
(**A**) Basal GCase activity of WT, p.N370S and p.L444P *GBA1* mutant BE(2)M17 cell lines, results are presented as mean ± SD values after two-way ANOVA followed by Tukey’s multiple comparisons test, * *p* < 0.05, ** *p* < 0.001, **** *p* < 0.0001; (**B**) GCase activity assay of differentiated WT, N370S and L444P neuronal cell lines treated for four days with 25 μM compound **3**. Activity is expressed as fold activity versus vehicle in each cell line (dashed line); (**C**) Representative images of GCase immunodetection by western blot and (**D**) quantification of GCase protein levels (GCase protein levels in vehicle-treated cells are represented as a dashed line) of the three differentiated neuronal cell lines (WT, N370S, and L444P) treated with 25 µM compound **3** for ten days; (**E**) GCase activity assay of three differentiated neuronal cell lines (WT, N370S and L444P) treated for 10 days with 25 μM of the selected compound **3**. Activity is expressed as fold activity versus vehicle in each cell line (dashed line); (**F**) Quantification of GlcSph levels, the substrate of GCase, following treatment of 10 days with compound **3** at 25 µM. Lipid levels are expressed in pmol/mg of tissue; and (**G**) Viability assays for the three differentiated neuronal cell lines treated for 10 with compound **3** at 25 µM. Viability is expressed as a fold percentage of live cells versus vehicles in each cell line. Results in panel (**B**,**D**–**G**) are presented as mean ± standard deviation values; significance is shown within each cell line compared to their vehicle after two-way ANOVA followed by Sidak’s multiple comparisons test, * *p* < 0.05, ** *p* < 0.01, *** *p* < 0.001, **** *p* < 0.0001. GCase, glucocerebrosidase; GlcSph, glucosylsphingosine; WT, wild-type.

After optimizing the effects of compounds **2** and **3** in a cellular model using patient fibroblasts, compound **3** was selected for further in vitro validation in a neuronal model. Similar to our observations in fibroblasts, we initiated the assessment of GCase activity after four days of treatment in WT, N370S, and L444P *GBA1*-expressing BE(2)-M17 dopaminergic neuron-like cells. As expected, *GBA1* mutations p.N370S and p.L444P result in a significant decrease in GCase activity under basal conditions, providing a suitable model to study the effect of the chaperones (Figure 6A). Consistent with the findings in fibroblasts, compound **3** enhanced GCase activity across all three cell lines (Figure 6B). To evaluate the enhancement of GCase stability mediated by the compounds, we initially quantified GCase protein levels using western blot analysis (Figure 6C,D). As anticipated, treatment with compound **3** increased total GCase protein levels, indicating that the compound’s binding can stabilize the protein’s conformation. This stabilization is particularly beneficial for mutant proteins, which are often more unstable and prone to degradation in the ER. As depicted in Figure 6D, the compounds effectively increased total GCase levels across all cell lines, except for WT cells, where levels remained comparable to the vehicle-treated control.

Our neuronal model uses stable BE(2)-M17 dopaminergic-like neuronal cell lines that constitutively express two prevalent *GBA1* mutations, p.N370S and p.L444P [37]. These neuronal cell lines undergo post-mitotic differentiation and display a dopaminergic phenotype, neurite extension, and sphingolipid accumulation [37]. Therefore, they serve as an excellent model for evaluating the biological efficacy of therapeutic interventions, such as our PCs, aimed at restoring GCase activity in vitro. Subsequently, we induced differentiation of BE(2)-M17 cell lines into post-mitotic neuronal cells, a crucial step for investigating the effects of lysosomal system impairment and its consequences in terms of intracellular macromolecule accumulation [37,38]. Following differentiation, cells were treated for an extended period (10 days) with the selected compounds to analyze their impact on GCase stability, GCase activity, sphingolipid accumulation, and cell viability. Consistent with the observed increase in GCase levels, enzymatic activity assays showed a corresponding enhancement in GCase activity (Figure 6E). Analysis by LC/MS-MS of sphingolipid levels through the quantification of GlcSph [37] demonstrated that compound **3** effectively reduced accumulated sphingolipid levels in *GBA1* mutant cell lines (Figure 6F). Cell viability assessments conducted after 10 days of treatment with the compounds were designed to explore potential cytotoxic effects from prolonged treatment, as well as the impact of increased GCase activity and decreased sphingolipid levels on cell viability. As shown in Figure 6G, treatment with compound **3** improved cellular viability, confirming the positive effects of compound **3** on overall cell survival.

### 2.6. BBB Penetration

**Figure 7:**
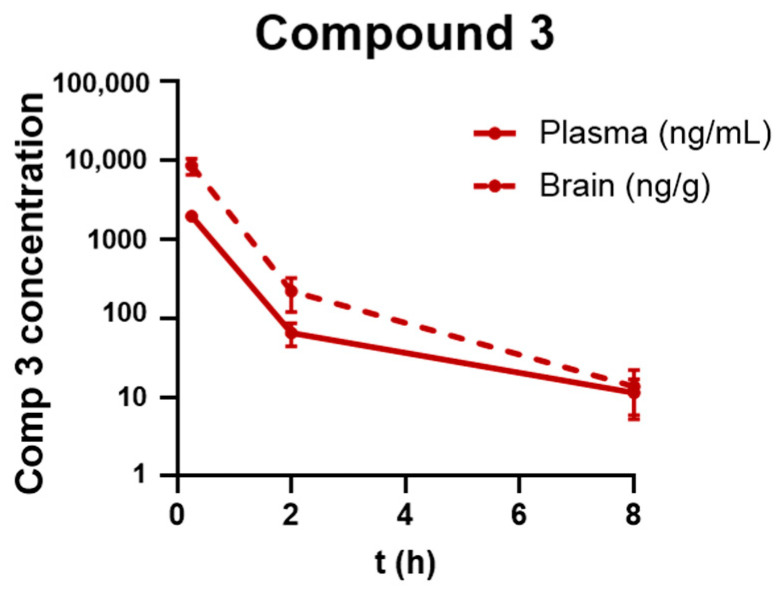
Plasma and brain pharmacokinetics distribution at different time points after administration of a single i.v. 10 mg/kg dose of compound **3** in male C57BL/6 mice.

Finally, we investigated whether compound **3** can cross the BBB and enter the central nervous system following intravenous (i.v.) administration. This is a preliminary step in developing the compound as a disease-modifying therapy for neuropathic GD and other neurological disorders linked to GBA mutations, such as PD and other synucleinopathies. Pharmacokinetics and brain penetrability were assessed by measuring the levels of compound **3** in brain tissue and plasma after intravenous administration (10 mg/kg) in wild-type mice. Figure 7 and Table 2 show that compound **3** has a favorable brain/plasma ratio, suggesting it can effectively penetrate the central nervous system (CNS).

## 3. Discussion

The discovery of small molecule stabilizers for GCase using SEE-Tx^®^ supercomputing technology has provided promising insights into potential PCs for the treatment of GD and related disorders. By leveraging the 3D structure of the GCase protein and advanced cutting-edge computational techniques, we identified previously undiscovered druggable allosteric sites. This innovative approach allowed us to convert these sites into molecular docking constraints, facilitating the virtual screening of vast compound libraries and selecting 179 promising hits.

Experimental testing using a thermal stabilization assay confirmed 28 of the compounds as effective stabilizers. Compound **1** emerged as the scaffold moiety of a new chemical series due to its binding to the GCase enzyme. This compound and its close analog, compound **2**, were characterized further, which eventually led to the design of new compounds with improved properties, such as compound **3**. Notably, compounds **2** and **3** increased GCase activity in patient-derived fibroblasts across various *GBA1* gene variants, demonstrating a dose-dependent effect. Both compounds showed binding affinity to GCase, as confirmed by SPR analysis, with compound **3** exhibiting favorable pharmacokinetics for potential CNS penetration. However, our study revealed that compound **2** also had unexpected inhibitory effects, emphasizing the complexities of designing PCs. While compound **2** inhibited GCase activity, compound **3** acted as a neutral chaperone, stabilizing the GCase protein without affecting its enzymatic function, thereby enhancing cellular activity. Further validation in neuronal models confirmed compound **3**’s potential by demonstrating increased GCase activity in dopaminergic-like cells, promising for treating neurological symptoms linked to GBA mutations. Moreover, compound **3** improved cell viability, suggesting its potential as a disease-modifying therapy for neuropathic GD and other neurodegenerative disorders such as PD.

Compound **3**’s ability to cross the blood-brain barrier and achieve significant brain penetration underscores its therapeutic potential for CNS disorders. Pharmacokinetic data showed a favorable brain/plasma ratio, supporting its future development as a CNS-targeted therapy.

While this study presents promising advancements, there are a few limitations to consider. One challenge was the unexpected inhibitory effects observed with compound **2**, highlighting the complexity of designing effective pharmacological chaperones. Additionally, the study was primarily conducted in vitro and in animal models, which may not fully replicate human physiological conditions. Future research should focus on expanding preclinical studies to include a wider range of models and eventually progress to clinical trials to better assess the safety and efficacy of compound **3** in humans. Furthermore, exploring the long-term effects and potential off-target interactions of compound **3** will be crucial in ensuring its viability as a therapeutic agent. Addressing these limitations will be essential to fully unlock the therapeutic potential of compound **3** for treating *GBA1*-associated disorders.

## 4. Materials and Methods

### 4.1. Virtual Screening Using SEE-Tx^®^ Technology

The published three-dimensional (3D) structure of human GCase, refined to a resolution of 1.95 Å, was used (PDB ID: 2V3F) [22]. Molecular dynamics simulations in organic-aqueous solvent mixtures (MDmix v0.1) revealed a druggable cavity [23,24,25,27] and identified key interaction sites, or binding hot spots, which were used as pharmacophoric restraints to guide docking and assess the binding site’s flexibility. A virtual library of approximately 5 million non-redundant chemical compounds from 5 vendors: Asinex (Moscow, Russia), Enamine (Kyiv, Ukraine), Life Chemicals (Kyiv, Ukraine), Princeton Biomolecular Research Inc. (Monmouth Junction, NJ, USA) and Specs (Zoetermeer, The Netherlands), along with compounds from our GAIN DB library, was computationally evaluated using the rDock 2013.1 program [28]. The evaluation employed a standard scoring function, pharmacophoric restraints, and a high-throughput protocol.

### 4.2. DSF

The ability of the compounds to stabilize the GCase protein was evaluated using DSF. This involved monitoring the thermal denaturation of the purified human native enzyme with the fluorescent probe SYPRO™ Orange (Thermo Fisher Scientific, Waltham, MA, USA), which binds to hydrophobic regions exposed during protein unfolding. Each reaction (25 μL) was conducted in triplicate in a 96-well plate. The reactions included 12.5 µL of 1.5 µM rhGCase protein (R&D Systems) in a buffer of 10 mM Na_3_PO_4_, 164 mM NaCl, pH 7.2, resulting in a final protein concentration of 1 µM with SYPRO Orange 10x. Additionally, 12.5 µL of various compound solutions, initially dissolved in 100% DMSO and diluted in the protein buffer to a final concentration of 1% DMSO, were added. The fluorescence intensity of SYPRO Orange was measured as the temperature increased using the Roche LightCycler^®^ 480 II (Roche Diagnostics). The significance of melting temperature (Tm) shifts was determined by two criteria: an absolute ∆Tm shift greater than 0.5 °C (instrumental criterion) and an absolute ΔTm standard deviation of 0.2 or less (statistical criterion). The thermal shift dose response was tested with GCase (R&D Systems) in two independent experiments, each performed in triplicate at a concentration of 30 µM for the DSF assays.

### 4.3. Enzyme Enhancement Activity

Fibroblasts from a healthy individual or patient-derived fibroblasts were obtained from the Coriell Institute for Medical Research (Camden, NJ, USA), Telethon Network of Genetic Biobanks (Roma, Italia), or Vall d’Hebron Research Institute (Barcelona, Spain) (Table 3).

The impact of the compounds on enhancing GCase enzymatic activity was evaluated using the following method: Patient-derived fibroblasts were seeded at 5000 cells per well in 96-well plates with Dulbecco’s Modified Eagle’s Media (DMEM), supplemented with 10% fetal bovine serum (FBS) and 1% penicillin/streptomycin (P/S) (Thermo Fisher Scientific, Waltham, MA, USA). The cells were incubated overnight at 37 °C with 5% CO_2_ to allow for attachment. The next day, the cells were treated with or without the specified compound at the given concentrations for four days. After treatment, the cells were washed with PBS and incubated with 5 mM 4-methylumbelliferyl-beta-D-glucopyranoside (Apollo Scientific, Cheshire, UK) in a 0.1 M acetate buffer at pH 4 for one hour at 37 °C. The reaction was halted by adding 200 µL of 100 mM Glycine-NaOH buffer at pH 10.7. The released 4-MU was measured using a Glomax^®^ Discover microplate reader (Promega, Madison, WI, USA), with excitation at 340 nm and emission at 460 nm.

Differentiated BE (2)-M17 cells were treated with either vehicle (0.25% DMSO) or compound **3** as specified, and then collected in lysis buffer containing 20 mM Tris (pH 7.5), 1 mM EDTA, 150 mM NaCl, 1% Triton, and freshly added protease inhibitors. Protein concentration was measured using the bicinchoninic acid (BCA) assay following the manufacturer’s instructions (Pierce^®^ BCA Protein Assay, Thermo Scientific). GCase activity was assessed in triplicate using 96-well plates with 10 µg of total homogenate samples. These samples were resuspended in McIlvaine’s buffer (0.2 M citrate/phosphate buffer, pH 5.4) containing 0.25% Triton X-100, 22 mM sodium taurocholate hydrate (SIGMA, 86339), and 5 mM of the fluorescent substrate 4-Methylumbelliferyl β-D-glucopyranoside (M363; Sigma-Aldrich, San Luis, MO, USA). A 100 μL sample per well was prepared, transferred to 96-well plates in triplicate, and incubated for at least 1 h at 37 °C. The reaction was stopped by adding 25 μL of 0.25 M glycine/NaOH (pH 10.4), and the released 4-methylumbelliferone was measured using an FLx800 spectrofluorimeter (BioTek, Winooski, VT, USA) with excitation at 365 nm and emission at 450 nm. Activity measurements were corrected based on the protein concentration determined in parallel using the BCA assay.

### 4.4. SPR Assays

All SPR experiments were performed at 20 °C using the Biacore T200 (GE Healthcare, Uppsala, Sweden). SPR detects changes in the refractive index on the sensor surface due to mass changes, which helps measure the interaction between the GCase protein (Cerezyme) and the tested compounds.

The human full-length GCase protein (Cerezyme, wild type, not tagged; Genzyme, Naarden, The Netherlands) was immobilized on an SPR CM5 sensor (29149603, GE Healthcare, Chicago, IL, USA) using standard amino coupling with a high protein concentration of 100 µg/mL. A nine-point, 2-fold serial dilution starting from 180 µM of the compound (from a 10 mM stock solution in DMSO) was tested by SPR at pH 7.4 in a buffer containing 10 mM Hepes, 5 mM EDTA, 150 mM NaCl, 0.01% Tween-20, 0.5 mg/mL CM-Dextran, and 5% DMSO. Reference channels included empty, activated, and deactivated channels on the same SPR sensor. The raw SPR signals from the active channel with immobilized GCase were adjusted by subtracting signals from the reference channel and the running buffer (double referenced) and corrected for any DMSO signal mismatch. Binding affinity values were extracted by fitting the SPR data to a four-parameter logarithmic dose-response equation without constraints using GraphPad Prism version 10.2.3.

### 4.5. GCase Biochemical Assay in Wild-Type Lysates

Lysates were prepared from wild-type fibroblasts using a lysis buffer containing 0.9% NaCl and 0.01% Triton-X100. Protein concentration was measured with a BCA protein assay kit. The cell lysates were then incubated with the specified compound for 15 min. The assay was conducted in a reaction buffer with 5 mM 4-methylumbelliferyl-beta-D-glucopyranoside in 0.1 M citrate-phosphate buffer at pH 5.6. The reactions were incubated at 37 °C for 1 h. To stop the reaction, 140 µL of 100 mM Glycine-NaOH buffer at pH 10.7 was added. The released 4-MU was measured using a Glomax^®^ Discover microplate reader, with excitation at 340 nm and emission at 460 nm.

### 4.6. Lysosomal GCase Activity Assay

Fibroblasts were seeded into a 12-well plate and treated with compounds for four days. After treatment, the fibroblasts were washed with PBS, and 320 µM PFB-FDGlu in PBS was added for 30 min at 37 °C. The cells were then washed with PBS and detached using trypsin. The trypsinization process was halted by adding cold PBS with 4% FBS. The cells were centrifuged at 1200 rpm for 5 min and washed twice with 200 µL of the previously mentioned buffer. The cell pellet was resuspended in 280 µL of FACS buffer containing DAPI (1:1000 dilution). Flow cytometric analysis was conducted using a Coulter Gallios I flow cytometer (492/516 nm), and the data were analyzed with FlowJo v10.8.1 software.

### 4.7. Lipid Quantification by LC-MS/MS

For fibroblast samples, cell pellets were dissolved in 150 µL of methanol containing 0.5% formic acid and 1 µM GalCer-d35 (C18 Galactosylceramide-d35 [d18:1/18:0-d35]; 24467 Cayman, Ann Arbor, MI, USA) as an internal standard. The samples were shaken for 1 h and sonicated once for 4 s at 30% amplitude. The supernatant was then collected by centrifugation at 13,000 G for 5 min at 4 °C. Sphingolipid analysis was performed using UPLC with an Agilent 1290 Infinity II/QQQ G6475 mass spectrometer system. An InfinityLab Poroshell 120 HILIC column (2.1 × 100 mm, 1.9 µm) was used at a flow rate of 0.5 mL/min. Solvent A was water with 5 mM ammonium formate and 0.1% formic acid, while solvent B was a mixture of acetonitrile, water, and methanol (95:2.5:2.5) with 5 mM ammonium formate and 0.1% formic acid. The gradient program was: 0–0.1 min at 95% B, 0.1–1.0 min from 95% to 0% B, 1.0–2.0 min at 0% B, 2.0–2.1 min from 0% to 95% B, and 2.1–2.5 min at 95% B. The mass spectrometer operated in positive ESI mode with a capillary voltage of 4600 V, nozzle voltage of 1700 V, gas temperature of 280 °C, gas flow of 10.5 L/min, and a nebulizer pressure of 33 psi. The sheath gas temperature was 270 °C with a flow of 11.5 L/min. HexCer was monitored in multiple reaction monitoring (MRM) mode, quantifying the 728.6 > 710.5 *m*/*z* transition.

For BE(2)-M17 samples, lipid extraction to quantify sphingolipids was conducted as outlined in Navarro-Romero et al. [37]. In brief, cultured cells were harvested, washed with PBS by centrifugation at 800× *g* for 5 min at 4 °C, and stored as dry pellets at −80 °C until processing. For lipid extraction, the frozen pellets were dissolved in 600 μL of solution W MC IS, which is methanol:chloroform (2:1) solution containing 0.01% butylated hydroxytoluene (BHT), 1 nM internal standard glucosyl(ß)-sphingosine-d5 (860636P Avanti Polar Lipids, Inc., Alabaster, Al, USA), and 13 nM C18 glucosyl(ß)-ceramide-d5 solution. The samples were vigorously vortexed for 1 min, left at room temperature for 30 min, and then sonicated twice for 15 s with a 10-s interval. The samples were centrifuged at 13,000 rpm for 5 min, and the supernatant was transferred to glass vials. The supernatant was evaporated to dryness under a stream of nitrogen and reconstituted with 500 µL of methanol:chloroform (1:2) before being transferred into chromatography vials for quantification via liquid chromatography tandem-mass spectrometry (LC-MS/MS). Total hexosylsphingosine (HexSph) analysis was performed using a Shimadzu LCMS-8050 at the Biochemistry Unit of Hospital Vall d’Hebron.

### 4.8. Protein Quantification by Western Blot

Cells were collected by trypsinization using Trypsin for 3 min at 37 °C and then centrifuged at 850× *g* for 5 min at 4 °C. The cell pellet was washed twice with PBS and resuspended in 40 µL of lysis buffer (20 mM Tris-HCl pH 7.5, 1 mM EDTA, 150 mM NaCl, 1% Triton X-100) with protease inhibitors at a 1/100 dilution. Protein concentration was measured using the bicinchoninic acid (BCA) assay following the manufacturer’s instructions (Pierce^®^ BCA Protein Assay, Thermo Scientific). Proteins (25 µg) were separated on SDS-PAGE gels with a 4% stacking gel and varying concentrations of running gel, then transferred to nitrocellulose membranes by electroblotting. Membranes were blocked with 5% milk in PBS, incubated with primary antibodies overnight at 4 °C, and then with secondary antibodies for 1 h at room temperature. Detection was performed using SuperSignal West Pico (Thermo Scientific) and visualized with the Odyssey LI-COR XF system. Bands were analyzed using ImageJ software 1.50a (NIH).

### 4.9. Cell Viability

Cells were plated and treated with compound **3,** as previously described. After completing the treatment, the viability of the differentiated cells was assessed by calculating the percentage of viable cells, which was determined by counting both viable and dead cells. Triplicate measurements from at least three independent samples were analyzed using a Muse Cell Analyzer cytometer. The analysis employed the Muse Count & Viability Assay Kit (MCH100102, Thermo Fisher), which stains viable and non-viable cells based on their permeability to two DNA-binding dyes included in the reagent.

### 4.10. NeuroPK

The brain penetration of compound **3** following intravenous injection was evaluated as follows. Nine male C57BL/6 mice received an intravenous dose of 10 mg/kg of the compound in a solution formulation consisting of 5% *v*/*v* NMP (N-methyl-2-pyrrolidone), 10% *v*/*v* Solutol^®^ HS-15, 30% *v*/*v* PEG-400, and 60% *v*/*v* normal saline. Blood samples (approximately 60 µL) were collected under light isoflurane anesthesia from three mice at each of the following time points: 0.25, 2, and 8 h. Plasma was obtained by centrifuging the blood and stored at −70 ± 10 °C until analysis. Brain samples were also collected from each mouse at the respective time points. Tissue samples were homogenized using ice-cold phosphate-buffered saline (pH 7.4), and the homogenates were stored below −70 °C until analysis. The total homogenate volume was three times the brain weight.

The concentration-time data from plasma and brain samples were used for pharmacokinetic analysis. Both plasma and brain samples were quantified using a fit-for-purpose LC-MS/MS method, with a lower limit of quantification (LLOQ) of 1.00 ng/mL for plasma and 2.00 ng/mL for the brain. The LC conditions were as follows: Thermo Scientific™ Accucore™ C18 column (2.7 µm, 50 mm × 2.1 mm), run time of 1.6 min, injection volume of 1 µL, and flow rate of 0.8 mL/min. The mobile phase consisted of 0.1% formic acid in acetonitrile (A) and 10 mM ammonium formate (B), with the following gradient: 0.00 min/95% B, 0.30 min/95% B, 0.50 min/5% B, 1.20 min/5% B, 1.40 min/95% B, and 1.60 min/95% B.

### 4.11. Data and Statistical Analysis

Statistical analysis was conducted using GraphPad Prism version 10.2.3, with data from at least three independent biological replicates. Most quantifications are expressed as a fold change compared to control or untreated conditions unless specified otherwise in the graphs.

## 5. Conclusions

Overall, this study illustrates the potential of compound **3** as a promising candidate for treating *GBA1*-related disorders by enhancing GCase function and demonstrating effective CNS delivery. By integrating computational innovation with rigorous experimental validation, the research marks a significant advancement in the development of PCs for these disorders. The study’s approach, which involves targeting the GCase protein with small molecules, not only enhances our understanding of GCase deficiency but also paves the way for new therapeutic strategies. This work lays a strong foundation for identifying and validating allosteric PCs, offering promising avenues for targeted therapies for GD and related conditions such as PD. To fully realize the therapeutic potential of compound **3**, further preclinical and clinical studies are crucial to understanding its impact on disease progression and patient outcomes.

## Figures and Tables

**Table 1:** Summary of SPR parameters.

Compound	2	3
Dose-response	Yes	Yes
K_D_ (µM)	29.8	50.7
Rmax (RU)	35.57	22.73
Offset (RU)	1.959	−1.406
Chi^2^ (RU^2^)	1.28	2.61

K_D_, dissociation constant; Rmax, maximum response; RU, resonance units; SPR, surface plasmon resonance.

**Table 2:** Summary of compound **3** concentrations in plasma (ng/mL) and brain tissue (ng/g) at the specified times post-administration.

Time (h)	Plasma Concentration (ng/mL)	Brain Concentration (n/g)	Brain-Kp
0.25	1967.97	8601.53	4.37
2	65.13	221.14	3.29
8	11.42	13.64	1.16

Kp, partition coefficient.

**Table 3:** Source of fibroblasts and corresponding genotype/phenotype.

Genotype	Phenotype	Source
wild-type (WT)	Healthy individual	GM03377, Coriell
p.L444P/p.L444P	Gaucher type II	GM08760, Coriell
p.N370S/84gg	Gaucher type I	GM00372, Coriell
p.N188S/p.S107L	Gaucher type II	20843, Telethon
p.L444P/p.WT	Parkinson	C.0006794, Vall d’Hebron Research Institute

## Data Availability

The protein structure used in this manuscript was downloaded from the Protein Data Bank (PDB IDs: 2V3F). The original contributions presented in the study are included in the article/Supplementary Material, further inquiries can be directed to the corresponding author/s.

## Supplementary Materials

The following supporting information can be downloaded at https://www.mdpi.com/article/10.3390/ijms26010009/s1.
