# Supplementary material for: Developing Allosteric Chaperones for GBA1-Associated Disorders—An Integrated Computational and Experimental Approach
Source: Int J Mol Sci. 2024 Dec 24;26(1):9. doi: 10.3390/ijms26010009 (PMC11720699; doi:10.3390/ijms26010009)
Supplement: Supplementary file 1 [file ijms-26-00009-s001.zip › ijms-3354744-supplementary.pdf]

Table S1. Thermal Shift Assay Results for Virtual Hits (179 compounds). This table presents the thermal shift (ΔTm) observed for wild-type recombinant human glucocerebrosidase (rhGCase, R&D Systems) in the presence and absence of 179 virtually screened compounds. Differential scanning fluorimetry (DSF) screening was performed in triplicate (n=1) at pH 7.2 with a compound concentration of 30 μM, with select hits undergoing a second independent triplicate experiment (n=2). The significance of melting temperature (Tm) shifts was evaluated using two criteria: an absolute ΔTm shift ≥ 0.5 °C (instrumental criterion) and an absolute ΔTm standard deviation ≤ 0.2 °C (statistical criterion).

Compound (Validated hit)	ΔTm (ºC)	S.D.	n
1	-0,16	0,11	1
2	-0,10	0,04	1
3	-0,64	0,11	1
4	-0,05	0,09	1
5	-0,36	0,05	2
6 (1)	3,30	0,13	2
7 (2)	1,55	0,05	2
8	-0,19	0,08	1
9 (3)	0,53	0,06	2
10	0,05	0,17	1
11	-0,12	0,07	1
12	0,00	0,05	1
13	0,02	0,01	1
14	-0,02	0,07	1
15	0,04	0,09	1
16	-0,01	0,05	2
17	-0,08	0,11	1
18	-0,05	0,18	1
19	-0,25	0,04	1
20	0,28	0,12	1
21	-0,13	0,08	1
22	0,32	0,04	1
23	0,01	0,02	1
24	-0,19	0,04	2
25	0,20	0,06	1
26	0,21	0,21	1
27	0,17	0,09	1
28	0,05	0,02	2
29	-0,14	0,05	1
30	0,04	0,02	1
31	-0,01	0,19	2
32	-0,03	0,05	1
33	-0,05	0,17	2

Compound (Validated hit)	ΔTm (ºC)	S.D.	n
34	0,25	0,05	1
35	0,15	0,13	2
36	0,07	0,06	1
37	0,20	0,21	1
38	-0,10	0,02	1
39	-0,32	0,12	1
40	0,12	0,23	1
41	-0,10	0,05	1
42	-0,23	0,06	1
43	0,10	0,18	2
44	0,00	0,03	1
45	0,04	0,06	1
46	0,35	0,07	2
47	0,26	0,03	1
48	0,26	0,12	2
49 (4)	1,52	0,10	2
50	0,23	0,04	1
51	0,05	0,02	1
52	0,09	0,04	1
53	0,31	0,06	2
54	0,13	0,01	1
55	0,09	0,06	2
56	0,28	0,11	2
57	0,13	0,08	1
58	0,04	0,18	1
59	0,03	0,11	1
60	0,02	0,13	1
61	-0,03	0,05	1
62	0,00	0,06	2
63	0,11	0,03	1
64 (5)	1,07	0,11	2
65	-0,11	0,08	1
66	0,01	0,08	1
67	0,39	0,08	2
68	-0,04	0,05	1
69	0,12	0,07	2
70	0,31	0,04	1
71	0,20	0,07	1
72	0,06	0,03	1
73	0,01	0,03	1
74	-0,14	0,48	1
75	0,38	0,06	2
76	0,24	0,11	1
77 (6)	0,62	0,09	2

Compound (Validated hit)	ΔTm (ºC)	S.D.	n
78	0,11	0,04	1
79	0,28	0,18	1
80	0,04	0,05	1
81	0,23	0,13	1
82	-0,12	0,16	2
83	0,10	0,12	1
84 (7)	4,09	0,09	2
85	0,35	0,04	2
86	0,08	0,02	1
87	0,09	0,12	1
88	0,10	0,19	1
89	0,00	0,09	1
90	0,18	0,13	2
91	0,30	0,09	2
92	0,48	0,06	2
93 (8)	1,10	0,10	2
94	-0,16	0,20	1
95	-0,16	0,06	1
96	-0,43	0,11	1
97	-0,17	0,12	1
98 (9)	0,50	0,17	1
99	0,12	0,15	2
100 (10)	1,77	0,07	2
101	0,02	0,03	1
102	0,39	0,07	2
103	0,13	0,02	2
104	0,06	0,11	2
105	0,13	0,11	2
106	0,17	0,06	2
107 (11)	1,59	0,08	2
108 (12)	0,55	0,06	2
109 (13)	0,51	0,08	2
110	0,19	0,04	1
111 (14)	0,52	0,11	2
112 (15)	0,71	0,05	2
113	0,11	0,11	2
114	0,38	0,14	2
115 (16)	0,63	0,20	2
116	-0,31	0,13	2
117	0,06	0,54	2
118	0,09	0,05	2
119	0,24	0,13	2
120 (17)	0,51	0,12	2
121	0,44	0,11	2

Compound (Validated hit)	ΔTm (ºC)	S.D.	n
122	-0,11	0,20	1
123	0,05	0,12	2
124	-0,09	0,04	2
125	-0,25	0,06	2
126	-0,39	0,14	2
127	0,07	0,08	2
128	0,25	0,06	2
129 (18)	1,53	0,12	2
130 (19)	0,69	0,06	2
131	0,08	0,06	1
132 (20)	1,09	0,03	2
133	-1,03	0,15	1
134	0,46	0,15	2
135 (21)	2,13	0,13	2
136	0,20	0,14	2
137	0,28	0,15	2
138	-0,23	0,18	1
139	-1,78	0,11	1
140	-0,17	0,13	1
141	-0,32	0,11	1
142	0,07	0,04	1
143 (22)	1,46	0,10	2
144	0,43	0,06	1
145	-0,31	0,09	2
146	0,19	0,12	2
147	0,12	0,04	1
148	0,43	0,06	2
149	-0,01	0,06	2
150	-0,07	0,03	1
151	0,34	0,11	1
152	-0,22	0,01	1
153	-0,48	0,06	1
154	-0,52	0,08	2
155	-0,05	0,07	2
156	-0,12	0,04	2
157	-0,05	0,08	2
158	-0,23	0,02	1
159 (23)	0,96	0,17	2
160	-1,17	0,24	1
161 (24)	0,87	0,08	2
162 (25)	0,78	0,06	2
163 (26)	0,54	0,10	2
164	-0,05	0,04	2
165	0,32	0,08	2

Compound (Validated hit)	ΔTm (ºC)	S.D.	n
166	0,14	0,05	1
167	0,02	0,24	1
168	0,24	0,07	2
169 (27)	0,67	0,14	2
170	-0,66	0,27	1
171	0,36	0,11	2
172 (28)	0,70	0,07	2
173	-0,55	0,10	2
174	0,28	0,12	2
175	-0,28	0,01	1
176	0,04	0,17	1
177	-0,51	0,02	1
178	0,11	0,03	2
179	0,19	0,16	2
